# Influence of Maternal Age on the Relationship Between Endometrial Thickness and Ongoing Pregnancy Rates in Frozen–Thawed Embryo Transfer Cycles: A Retrospective Analysis of 2,562 Cycles

**DOI:** 10.3389/fendo.2022.821753

**Published:** 2022-04-27

**Authors:** Haiqing Tian, Hejiang Zhang, Hong Qiu, Xuejiao Yang, Xiaolin La, Lei Cui

**Affiliations:** ^1^ First Affiliated Hospital of Xinjiang Medical University, Urumqi, China; ^2^ School of Life Science and Technology, Xi’an Jiaotong University, Xi’an, China; ^3^ Reproductive Medicine Center, First Affiliated Hospital of Xinjiang Medical University, Urumqi, China

**Keywords:** endometrial thickness, FET, ongoing pregnancy rates, cleavage stage embryos, blastocysts

## Abstract

**Background:**

In frozen–thawed embryo transfer (FET) cycles, endometrial thickness (EMT) has been used routinely as the main clinical monitoring index. However, the current findings are conflicting.

**Method:**

This was a single-center retrospective study of 2,054 couples (2,562 cycles) who underwent FET (including cleavage stage embryos and blastocysts) between January 2017 and August 2020 in the reproductive centers of First Affiliated Hospital of Xinjiang Medical University. The primary outcome measure was the ongoing pregnancy rate (OPR); the secondary outcome was the clinical pregnancy rate.

**Results:**

After stratified analysis and adjusting for confounders such as maternal age, duration of infertility, number of high-quality embryos transferred, endometrial preparation protocol, number of transfer cycles, and stages of embryo transferred, we found a curvilinear relationship between EMT and the OPR in women < 35 years of age. For women with EMT ≤ 8 mm, the OPR increased by 150% for cleavage stage embryo transfer for every 1 mm increase in the EMT; similarly, it increased by 97% for blastocyst stage FET. However, there was a linear relationship between EMT and OPR in women aged ≥ 35 years. When blastocysts were transferred, for every 1 mm increase in the EMT the OPR increased significantly by 12%. But OPR after frozen-thawed cleavage stage embryos transfer did not increase significantly with increased EMT

**Conclusions:**

Our study showed that the OPR increased significantly with increased EMT between young women aged < 35 years with EMT ≤ 8 mm and older women who underwent transfer of blastocysts.

## Introduction

In recent years, assisted reproductive technology (ART) has been applied worldwide. Although fresh embryo transfer is a clinical routine, frozen–thawed embryo transfer (FET) is becoming increasingly popular, mainly because it can help prevent ovarian hyperstimulation syndrome (OHSS). Embryos can be better implanted in natural cycles to avoid the negative impact of ovarian hyperstimulation (COH) on endometrial receptivity. This could help optimize the cumulative live birth rate in a single cycle. Considering the relatively limited success rate with corresponding costs, it would be interesting to assess the factors affecting pregnancy outcomes in FET and improve pregnancy rates.

The use of transvaginal ultrasound for measuring endometrial thickness (EMT) in ART is well established. Recording the EMT has become part of standard monitoring in ART, mainly because ultrasonography is simple, noninvasive, reproducible, and is the ideal tool for this task (1, 2). EMT is the most common and important indicator used for endometrial receptivity and potentially can predict the success of ART (3–5). Therefore, it is used as an indicator for clinical routine judgment of whether endometrial development is sufficient to determine the timing of embryo transfer. However, there is considerable controversy regarding EMT in ART (6, 7).Two recent large meta-analyses on the relationship between EMT and pregnancy outcomes have shown inconsistent results. This is because of heterogeneity in study populations and because different cut-off points of EMT have been used in various studies. The independent significance of EMT remains to be further investigated in ART procedures. We investigated whether EMT would affect the ongoing pregnancy rate (OPR) when cleavage stage embryos or blastocysts were transferred by FET in different age groups. This should provide a reference for decision-making in ART clinics.

## Materials And Methods

### Study Design

This was a single-center retrospective observational study with a protocol approved by the hospital’s ethics committee (permit number k202103-05). All couples provided written informed consent before treatment, and data were entered prospectively into a computerized database. We included 2754 FET cycles (including cleavage stage embryos and blastocysts) between January 1, 2017 and August 31, 2020. The exclusion criteria were as follows (1): congenital uterine malformations, such as unicornuate, bicornuate, didelphys, or septate uteri as diagnosed by hysteroscopy or laparoscopy (2); patients with recurrent pregnancy loss; and (3) missing key data on the day of ovulation or FET. Finally, 2562 cycles in 2054 couples were included for analysis. The causes of infertility were divided into tubal factors, endometriosis, abnormal ovulation, male factors, and unexplained.

### Treatment Procedures

All participants were treated according to routine ART procedures, and controlled ovarian hyperstimulation (COH) and oocyte retrieval have been described widely in the literature. Recombinant follicle stimulating hormone (Gonal-F; Merck-Serono) and human menopausal gonadotropin were used for COH. Protocols were selected according to the patient’s documented ovarian reserve. These included an early follicular phase long-acting gonadotropin-releasing hormone (GnRH) agonist long protocol, a GnRH antagonist protocol, mid-luteal-phase short-acting GnRH-agonist long protocol, natural protocol, mild stimulation protocol, and progestin-primed ovarian stimulation. The final maturation of oocytes was induced by 5,000–10,000 IU of human chorionic gonadotropin (hCG), and oocytes were retrieved transvaginally at 36 h. Embryos were evaluated for quality at the time of FET according to the Istanbul embryo assessment consensus (8). Laboratory procedures and conditions were unchanged during this period.

### Endometrial Preparation Protocol

FET was performed in patients with a normal uterine cavity guided by ultrasonography. If endometrial or pelvic abnormalities were suspected, such as intrauterine adhesions, endometrial polyps, submucosal fibroids, uterine fluid or Fallopian tube effusion, appropriate diagnosis and treatment by hysteroscopy or laparoscopy were required from 1 month to 6 months before FET. This was performed using natural cycle (NC) or hormone replacement therapy (HRT). The NC approach was used for patients with normal ovulation, and HRT was applied according to each patient’s requirement, particularly for those with irregular menstrual cycles.

### Natural Cycle

Follicle sizes and the EMT were measured using transvaginal ultrasonography (TVU) every other day or daily from day 10 of the menstrual cycle. When the mean diameter of the dominant follicle was > 17 mm, the decision to trigger ovulation with 10,000 IU of hCG was made, based on serum hormone levels. The day of ovulation was defined as D0, and vaginal progesterone gel was administered until 10 weeks of gestation if the woman was pregnant. Cleavage stage embryos and blastocysts were transferred on days 3 and 5, respectively.

### Hormone Replacement Therapy

Serum basal hormone levels and uterine ultrasonography were checked on days 2 or 3 of the menstrual cycle. If the results were normal, oral estradiol valerate at 4 mg/day was started and increased to 6 mg/day on day 6. After 10 days of treatment, the endometrium was examined by TVU. If the EMT was ≥8 mm and there was no sign of a dominant follicle or ovulation, the same dose of estradiol was continued for 4 days, followed by ultrasonographic measurement of EMT on the same day. If the EMT was < 8 mm, the dose of estradiol was increased by vaginal or oral routes, and the EMT was monitored every other day. Estrogen therapy was not continued for more than 21 days for intimal transformation. Progesterone administration was started on D0 with oral dydrogesterone 10 mg twice daily, and vaginal gel (Crinone) was applied at 90 mg per day. The use of estradiol was tapered off when the patient had a positive hCG test. The use of Crinone vaginal gel continued until a fetal heartbeat was detected by TVU, and dydrogesterone was stopped at pregnancy day 10.

### Gonadotropin-Releasing Hormone Agonist Combined with Hormone Replacement Therapy (GnRHa-HRT)

GnRHa (3.75 mg) was injected during the menstrual period, and the process was similar to the above HRT cycle after 1 month.

### Measurement of EMT

EMT was measured uniformly using TVU performed by three trained reproductive specialists with a standardized protocol. They each had more than 10 years of experience using the same protocol. On the day of ovulation or the day of progesterone administration, EMT was defined as the thickest echogenic area from one stratum basalis endometrial interface across the endometrial canal to the other stratum basalis interface (9). The measurement accuracy was 0.1 mm and was recorded in our database. Most patients will have multiple uterine contractions every minute, and these can cause changes in the EMT of up to 3–4 mm. To ensure the most accurate measurement, protocol was followed strictly, waiting for the wave to pass before measurement. In our center, routine TVU of the endometrium is performed at least twice before embryo transfer, and patients with thin endometrium will be subjected to increased frequency of detection and drug treatment are used i to achieve the ideal endometrial status. The ultrasound equipment and measurement technology in our center was of good quality to reduce errors in EMT measurement values.

### Embryo Transfer

FET was performed under abdominal ultrasound guidance with a Cook catheter according to standard operating procedures. According to the specifications of our center, one or two embryos (occasionally three) were transferred into the uterine cavity. All frozen embryos were thawed on the day of transfer.

### Outcome Measures

The primary outcome was the ongoing pregnancy rate (OPR), defined as a live fetus identified by ultrasound on day 12. The secondary outcome was the CPR, defined as an intrauterine gestational sac with a fetal heart rate detected by ultrasonography 2–3 weeks after a positive hCG pregnancy test.

### Statistical Methods

Continuous variables are expressed as the mean ± standard deviation (SD). Categorical variables are expressed as the frequency or percentage. Differences among groups were compared using single-factor analysis of variance and chi-square tests. Variables in skewed distribution were tested using the Kruskal–Wallis method. A smoothing function was applied to the EMT and OPR data. The turning point was determined and provided the maximum likelihood model. The confounders were controlled for in two different models. Model 1 was adjusted for the following potential confounders: maternal age, endometrial preparation protocol, duration of infertility, number of good quality embryos transferred. Model 2 was further adjusted for the following covariables associated with OPR: maternal age, number of FET cycles, stage of transferred embryos.

**Figure 2:**
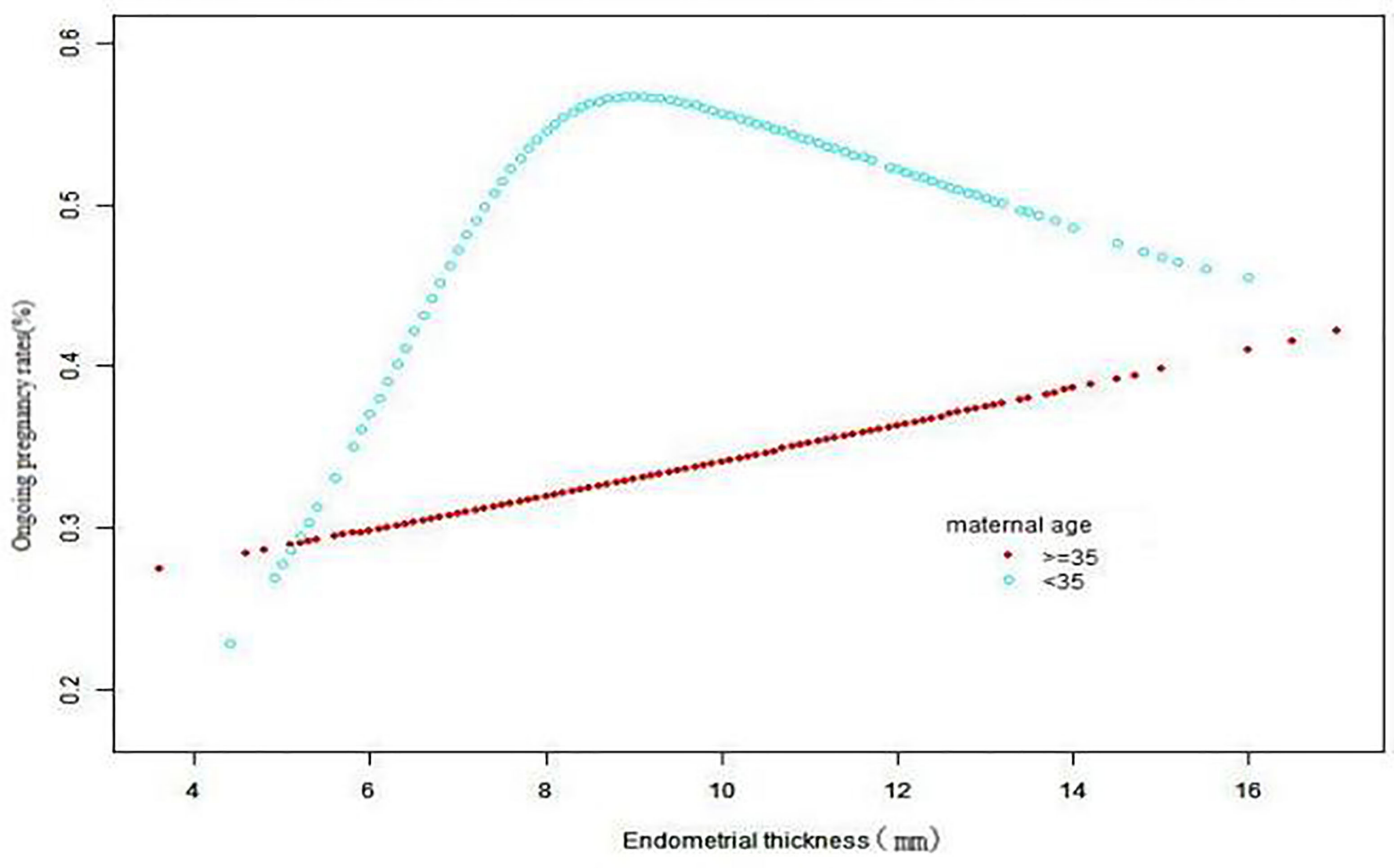
The association between EMT and OPR in two groups of women. A nonlinear relationship for women < 35 years and a linear relationship for women ≥35 years were detected after adjusting for endometrial preparation protocol, duration of infertility, maternal height, number of good-quality embryos transferred, number of transferred cycles, and stage of embryo transferred.

Data analyses were conducted on three levels (1): correlation between EMT and OPR (linear or non-linear) (**Figure 2**) (2); factors that might affect the correlation between EMT and OPR (3); correlation between EMT and OPR after adjustment for interfering factors or after stratified analysis. We applied a generalized additive model (GAM) to estimate the independent relationship of association between EMT and OPR (**Tables 3**, **4**). A smoothing linear regression GAM model was adjusted using R software to test any association between EMT and OPR (10, 11). The results are displayed graphically. The statistical software package R (http://www.R-project.org, the R Foundation) and Empower (R) (www.empowerstats.com, X&Y Solutions, Inc. Boston, MA, USA) were used for data analyses. Two-way P values < 0.05 indicated statistical significance. Results are presented as the odds ratio (OR) and 95% confidence interval (CI).

## Results

### Patient Baseline Data

**Figure 1:**
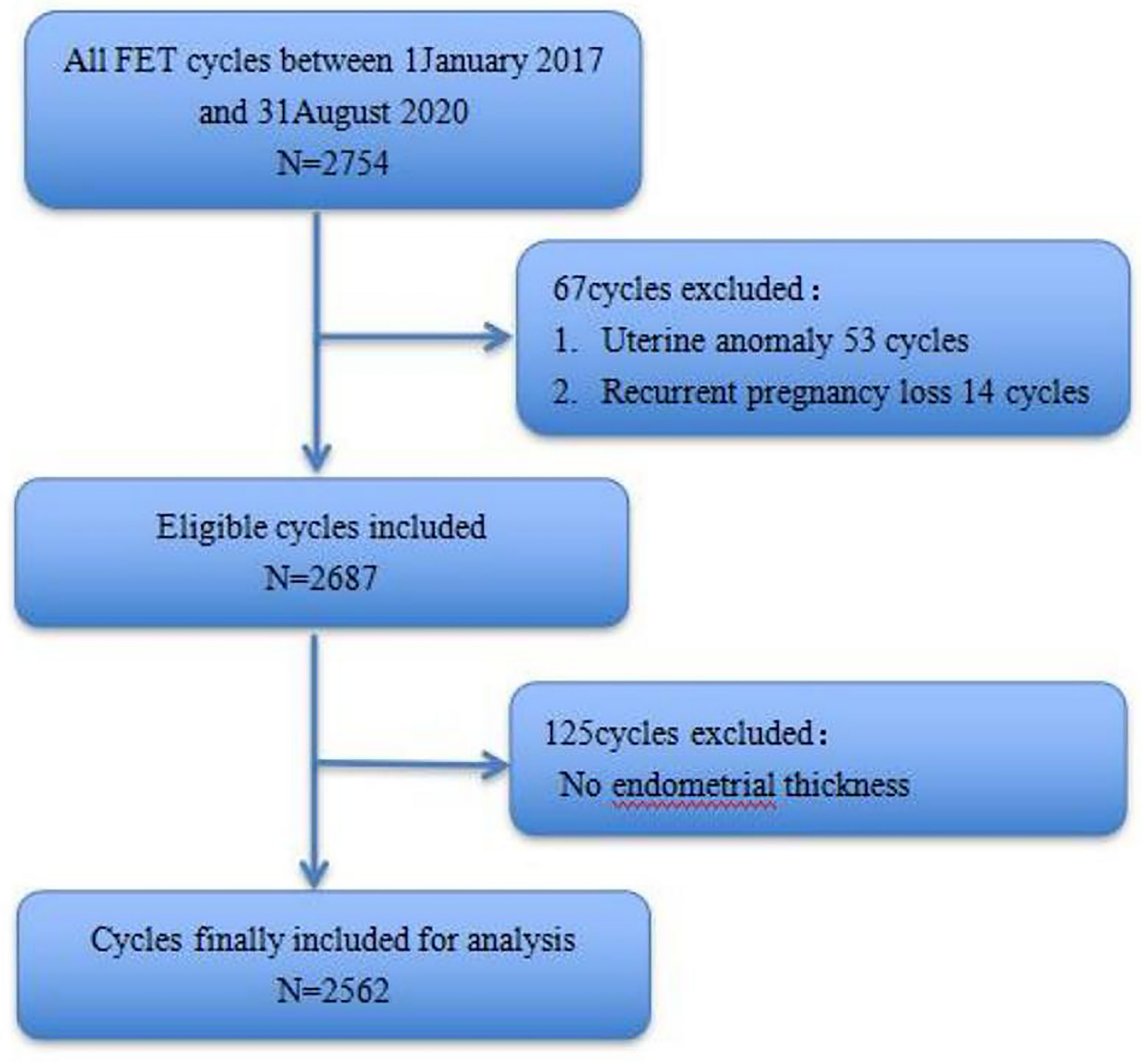
Flow chart of the patients enrolled and the study outline.

In all, 2754 FET cycles were recorded in our database and 192 cycles were excluded according to the criteria listed above (**Figure 1**). Finally, we included 2562 cycles in 2054 couples for analysis. For women aged < 35 years, there were 1531 cycles (59.76% of the total), and for women aged ≥ 35 years, there were 1031 cycles (40.24%). They were grouped according to EMT with a turning point of 7.7 mm. For the group with EMT≤ 8 mm, there were 522 cycles (20.37%), and for those with EMT > 8 mm, there were 2040 cycles (79.63%). For these two groups, the CPRs were 49.62% (259/522) and 55.59% (1134/2040), and the OPRs were 40.8% (213/522) and 47.21% (963/2040), respectively (**Table 1**).

**Table 1:** Baseline characteristics of the participants.

Endometrial thickness	< = 8	>8	Standardize diff.	P-value
No. of participants	522	2040		
maternal age (years)	34.61 ± 4.93	33.68 ± 4.73	0.19 (0.10, 0.29)	<0.001
paternal age (years)	36.45 ± 5.51	35.70 ± 5.34	0.14 (0.04, 0.24)	0.004
Duration of infertility (years)	3.86 ± 2.84	4.12 ± 2.70	0.09 (-0.00, 0.19)	0.003
gravidity	1.15 ± 1.34	0.75 ± 1.09	0.32 (0.23, 0.42)	<0.001
parity	0.11 ± 0.33	0.10 ± 0.34	0.02 (-0.07, 0.12)	0.624
maternal BMI (kg/m2)	22.67 ± 2.98	22.96 ± 3.15	0.09 (-0.00, 0.19)	0.060
hight of maternal (mm)	161.60 ± 4.50	162.17 ± 5.07	0.12 (0.02, 0.22)	0.019
weight of maternal (kg)	59.28 ± 8.51	60.44 ± 9.06	0.13 (0.04, 0.23)	0.008
hight of paternal (mm)	174.47 ± 5.36	174.26 ± 5.53	0.04 (-0.06, 0.14)	0.437
weight of paternal (kg)	78.16 ± 11.77	78.42 ± 12.07	0.02 (-0.08, 0.12)	0.663
paternal BMI (kg/m2)	25.63 ± 3.36	25.76 ± 3.41	0.04 (-0.06, 0.14)	0.426
No. of good quality embryos transferred	1.43 ± 0.59	1.36 ± 0.65	0.10 (0.01, 0.20)	0.093
No. of embryos transferred	1.69 ± 0.48	1.69 ± 0.48	0.01 (-0.09, 0.10)	0.887
EMT (mm)	7.21 ± 0.79	10.16 ± 1.43	2.55 (2.43, 2.67)	<0.001
No. of transferred cycles	1.43 ± 0.95	1.30 ± 0.72	0.15 (0.06, 0.25)	<0.001
endometrial preparation protocol			0.31 (0.22, 0.41)	<0.001
No.of natural cycle	122 (23.46%)	335 (16.59%)		
No.of HRT cycle	63 (12.12%)	114 (5.65%)		
No.of GnRHa-HRT cycle	335 (64.42%)	1570 (77.76%)		
Type of infertility			0.26 (0.16, 0.36)	<0.001
No.of primary infertility	230 (44.06%)	1161 (56.91%)		
No.of secondary infertility	292 (55.94%)	879 (43.09%)		
tubal factor infertility			0.01 (-0.09, 0.10)	0.883
0	220 (42.23%)	867 (42.58%)		
1	301 (57.77%)	1169 (57.42%)		
endometriosis			0.08 (-0.01, 0.18)	0.070
0	489 (93.86%)	1950 (95.73%)		
1	32 (6.14%)	87 (4.27%)		
anovulation			0.10 (0.01, 0.20)	0.034
0	333 (63.92%)	1401 (68.78%)		
1	188 (36.08%)	636 (31.22%)		
male factor infertility			0.02 (-0.07, 0.12)	0.638
0	395 (75.82%)	1524 (74.82%)		
1	126 (24.18%)	513 (25.18%)		
unexplained			0.10 (0.01, 0.20)	0.043
0	494 (94.82%)	1879 (92.24%)		
1	27 (5.18%)	158 (7.76%)		
Stimulation protocol			0.16 (0.06, 0.25)	0.103
Natural protocol	0 (0.00%)	7 (0.34%)		
EFLL protocol	282 (54.02%)	1222 (59.96%)		
GnRH antagonist	79 (15.13%)	259 (12.71%)		
MLSL protocol	39 (7.47%)	127 (6.23%)		
PPOS	113 (21.65%)	400 (19.63%)		
mild stimulation protocol	9 (1.72%)	23 (1.13%)		
Fertilization method			0.10 (0.01, 0.20)	0.038
IVF	379 (72.61%)	1385 (67.89%)		
ICSI	143 (27.39%)	655 (32.11%)		
stage of embryo transferred			0.05 (-0.04, 0.15)	0.283
cleavage	200 (38.31%)	730 (35.78%)		
blastocyst	322 (61.69%)	1310 (64.22%)		
Clinical pregnancy rates			0.12 (0.02, 0.22)	0.015
0	263 (50.38%)	906 (44.41%)		
1	259 (49.62%)	1134 (55.59%)		
maternal age			0.16 (0.06, 0.25)	0.001
>=35	242 (46.36%)	789 (38.68%)		
<35	280 (53.64%)	1251 (61.32%)		

EMT, endometrial thickness; BMI, body mass index; IVF, in vitro fertilization; ICSI, intracytoplasmic sperm injection; FET, frozen–thawed embryo transfer; EFLL, early-follicular-phase long-acting GnRH-agonist long protocol; MLSL, mid-luteal-phase short-acting GnRH-agonist long protocol; PPOS, Progestin-primed ovarian stimulation; HRT, hormone replacement therapy; GnRHa-HRT, gonadotropin-releasing hormone agonist combined with hormone replacement therapy.

Values are presented as the mean ± SD or number and (%).

### Relationship Between EMT and Ongoing Pregnancy Rate

After adjusting for confounders such as age, duration of infertility, number of high-quality embryos transferred, endometrial preparation protocol, number of transferred cycles, and stage of embryo transfer, EMT was analyzed by curve fitting with the OPR according to age groups.

### Results for Women Aged <35 Years

A nonlinear relationship was found between EMT and OPR in women aged < 35 years. The turning point of the EMT was 7.7 mm (**Table 2**). For women with an EMTs < 7.7 mm, the OPR increased significantly by 124% when the EMT increased by 1 mm, (OR 2.24; 95% CI 1.57–3.19; P < 0.0001). Further sensitivity analysis was performed. Grouping by cleavage stage embryos or blastocysts and adjusting for potential confounders that may be associated with OPR by Models I and II in multiple regression analysis showed that for EMT ≤ 8 mm, transfer of cleavage stage embryos and blastocysts and an increase in EMT of 1 mm, the OPR increased by 150% and 97%, respectively (OR 2.5; 95% CI 1.11–5.62; P = 0.026; and OR 1.97 95% CI 1.29–3.00; P = 0.0016, respectively; **Table 3**).

**Table 2:** Threshold effect analysis of EMT and OPR using linear regression analysis in women < 35 years of age.

Inflection point of EMT	Effect size (β)	95%CI	P value
<7.7	2.24	1.57 to 3.19	<0.0001
≥7.7	0.94	0.87 to 1.01	0.0904

Effect: OPR Cause: EMT.

Adjusted for maternal age, maternal height, duration of infertility, number of good-quality embryos transferred, endometrial preparation protocol, number of transferred cycles, stage of embryo transferred.

**Table 3:** Relationship between EMT and OPR in the two different models for women aged < 35 years or with EMT ≤ 8 mm.

Stage Of Embryo Transferred	Non-adjusted	Adjust I	Adjust II
cleavage	2.05 (1.00, 4.19) 0.0492	2.35 (1.07, 5.14) 0.0332	2.50 (1.11, 5.62) 0.0266
blastocyst	1.97 (1.33, 2.93) 0.0007	2.00 (1.32, 3.02) 0.0011	1.97 (1.29, 3.00) 0.0016
Total	1.99 (1.41, 2.81) <0.0001	2.06 (1.43, 2.95) <0.0001	2.05 (1.42, 2.95) 0.0001

β value (95% CI) P value / OR (95%CI) P value. Non-adjusted model.

Model I was adjusted for maternal age; endometrial preparation for FET; duration of infertility; and the number of good-quality embryos transferred.

Model II was adjusted for maternal age; maternal height; duration of infertility; number of good-quality embryos transferred; endometrial preparation protocol; and number of FET cycles.

When the EMT was > 7.7 mm, there was a 0.06% decrease in OPR for every 1 mm increase in the EMT (**Table 2**), but EMT was not significantly correlated with the OPR (OR 0.94; 95% CI 0.87–1.01; P = 0.0904).

### Results for Women Aged ≥ 35 years

In women aged ≥ 35 years with a total of 1031 cycles (530 with blastocysts and 501 with cleavage-stage embryos), the EMT was correlated linearly with the OPR. When blastocysts were transferred, for every 1 mm increase in the EMT the OPR increased significantly by 12% (OR 1.12; 95% CI 1.01–1.24; P = 0.0336). However, when cleavage stage embryos were transferred, changes in the EMT and OPR were not significantly correlated (OR 1.02; 95% CI 0.91–1.14; P = 0.7354; **Table 4**).

**Table 4:** Relationship between EMT and OPR in women ≥35 years of age in two different models.

Stage of Embryo Transferred	Non-adjusted	Adjust I	Adjust II
cleavage	1.03 (0.93, 1.15) 0.5237	1.04 (0.93, 1.15) 0.5187	1.02 (0.91, 1.14) 0.7354
blastocyst	1.08 (0.99, 1.19) 0.0949	1.11 (1.00, 1.23) 0.0404	1.12 (1.01, 1.24) 0.0336
Total	1.06 (0.99, 1.14) 0.0947	1.07 (0.99, 1.15) 0.0795	1.06 (0.99, 1.14) 0.1018

## Discussion

Our study is the first to reveal that EMT is associated with OPR in only some FET cycles. It explains the conflicting results of previous studies regarding EMT as a marker of endometrial receptivity and as a predictor of the success of ART treatment.

The largest study (12) to date on EMT and pregnancy outcomes, including 20,114 FET cycles, suggested that the CPR and live birth rates decreased when endometrial thickness was less than 7 mm. The study included all women undergoing FET, but did not exclude the role of other confounding factors besides EMT that could have had an impact on pregnancy outcome.

It is well known that the woman’s age is a negative factor affecting pregnancy outcomes after FET. One study (13) found that the duration of infertility, EMT before frozen embryo transfer, and the number of embryos transferred were three independent predictors of live birth rate after cleavage stage frozen embryo transfer in young women. In addition, Vaegter et al. (14) showed that a woman’s age was a predictor of live birth after ART. Maternal age was similarly shown to be a factor influencing OPR after FET in our study. Therefore, here the confounding factors including woman’s age, height, duration of infertility, embryo transfer protocol, numbers of high-quality embryos transferred, and numbers of FET cycles were determined by screening and by adjusting the variables individually.

After adjusting for confounders, our study showed a curvilinear relationship between EMT and OPR in young women (< 35 years old). The cut-off point of the EMT was 7.7 mm; thus, when the EMT was < 7.7 mm, the OPR increased by 124% when EMT increased by 1 mm. When the EMT was > 7.7 mm, there was no significant correlation between EMT and OPR.

In older women (age ≥ 35 years), we found a linear relationship between EMT and OPR. When frozen blastocysts were transferred, EMT was positively and significantly correlated with OPR, which increased by 12% for every 1 mm increase in EMT; however, there was no significant positive relationship between EMT and OPR after frozen cleavage stage embryos were transferred. This is mainly because it is the euploidy of embryos rather than EMT that determines their OPR after frozen cleavage stage embryos are used for FET in such women. Maternal age is the most important factor for the success of *in vitro* fertilization (15), and this is associated with embryo euploidy. Another study (16) also found that constant increases in chromosomal abnormalities with maternal age in frozen cleavage-stage ET. However, the chromosomal abnormality rates of the patients aged <35 years and those aged ≥35 years who underwent frozen blastocyst transfers were comparable. The inclusion of 13 RCT studies (17) found low quality evidence of live births and moderate quality evidence of clinical pregnancies suggesting that fresh blastocyst stage transfers have a higher rate than fresh cleavage stage transfers. A recent study (18) found that the EMT did not predict live birth in either fresh or FET cycles. A total of 287 FET cycles were included in the study, and the EMT values were 8.4 mm (range 7.4–9.7) and 9 mm (8, 19, 20) mm in women with and without live births, respectively (P = 0.38). Only 11.8% (66/560) of the study population had an EMT < 7 mm. However, the number of patients with inadequate endometrium in that study was small and this might have caused bias. A total of 522 cycles (20.37%) with EMT ≤ 8 mm were included in our study (**Table 1**). It is important to note in order to reduce bias, sensitivity analyses were conducted compared with prior published studies. Our study showed that the OPR increased significantly with increased EMT between young women aged < 35 years with EMT ≤ 8 mm and older women who underwent transfer of blastocysts.

Multiple studies have shown that among women undergoing embryo transfer, the actual numbers of those with insufficient endometrium are underestimated, because clinicians or patients may be more reluctant to perform embryo transfer, and cancelled cycles were not included. This group of women should receive more attention in ART clinics because they will tend to have more cycles cancelled or require multiple cycles of treatment. This will prolong the time to achieve a live birth and will involve more economic burden. Our study will provide guidance for clinical practice, showing that specific populations with insufficient endometrial thickness will have significantly improved pregnancy outcomes if the EMT can be increased.

### Advantages and Limitations

Through sensitivity analysis, this study has revealed for the first time why there have been conflicting results between EMT and pregnancy outcomes in previous studies. Thus, in these FET cycles, EMT was significantly associated with the OPR only in young women with an EMT ≤ 8 mm and older women who underwent transfer of blastocysts. This study provides a reference for clinical transplantation strategies and better counseling and advice for patients.

This study was limited by its retrospective design, and the mechanisms underlying these associations need to be confirmed by prospective studies with larger samples. However, such prospective studies are likely to be difficult to implement in ART clinics because clinicians and patients are reluctant to perform FET when the endometrium is insufficient. This study was conducted in FET cycles, and it is unclear whether it is applicable to fresh embryo transfer cycles. Moreover, the EMT on the day of FET was not recorded in this study, as transabdominal ultrasound measurements would have been required on the day of transplantation and these are not as accurate as TVU.

### Key Message

Through sensitivity analysis, this study has revealed for the first time why there have been conflicting results between EMT and pregnancy outcomes in previous studies. Thus, in these FET cycles, EMT was significantly associated with the OPR only in young women with an EMT ≤ 8 mm and older women who underwent transfer of blastocysts. This study provides a reference for clinical transplantation strategies and better counseling and advice for patients.

## Data Availability Statement

The original contributions presented in the study are included in the article/supplementary materials, further inquiries can be directed to the corresponding author/s.

## Ethics Statement

The studies involving human participants were reviewed and approved by Ethics Committee of First Affiliated Hospital of Xinjiang Medical University. The patients/participants provided their written informed consent to participate in this study.

## Author Contributions

HT, XL, and LC were responsible for the concept and study design. HZ contributed to statistical analysis. LC and XL contributed to revising the article. HT, HZ, HQ, and XY contributed to data collection. HT drafted the manuscript. All authors contributed to the critical discussion of the data and final editing of the manuscript. All authors read and approved the final manuscript.

## Funding

This work was financially sponsored by Natural Science Foundation of Xinjiang (NO. 2021D01C296).

## Conflict of Interest

The authors declare that the research was conducted in the absence of any commercial or financial relationships that could be construed as a potential conflict of interest.

## Publisher’s Note

All claims expressed in this article are solely those of the authors and do not necessarily represent those of their affiliated organizations, or those of the publisher, the editors and the reviewers. Any product that may be evaluated in this article, or claim that may be made by its manufacturer, is not guaranteed or endorsed by the publisher.

## References

[1] Delisle MF Villeneuve M Boulvain M. Measurement of Endometrial Thickness With Transvaginal Ultrasonography: Is it Reproducible? J Ultrasound Med (1998) 17(8):481–4. doi: 10.7863/jum.1998.17.8.481 9697950

[2] De Geyter C Schmitter M De Geyter M Nieschlag E Holzgreve W Schneider HP. Prospective Evaluation of the Ultrasound Appearance of the Endometrium in a Cohort of 1.186 Infertile Women. Fertil Steril (2000) 73(1):106–13. doi: 10.1016/s0015-0282(99)00484-7 10632422

[3] Momeni M Rahbar MH Kovanci EA. Meta-Analysis of the Relationship Between Endometrial Thickness and Outcome of *In Vitro* Fertilization Cycles. J Hum Reprod Sci (2011) 4:130–7. doi: 10.4103/0974-1208.92287 PMC3276947 22346080

[4] Lv H Li X Du J Ling X Diao F Lu Q. Effect of Endometrial Thickness and Embryo Quality on Live-Birth Rate of Fresh IVF/ICSI Cycles: A Retrospective Cohort Study. Reprod Biol Endocrinol (2020) 181(1):18:89. doi: 10.1186/s12958-020-00636-6 PMC7441697 32825835

[5] Tomic V Kasum M Vucic K. Impact of Embryo Quality and Endometrial Thickness on Implantation in Natural Cycle IVF. Arch Gynecol Obstet (2020) 05:3015(5). doi: 10.1007/s00404-020-05507-4 PMC7181434 32211954

[6] Nishihara S Fukuda J Ezoe K Endo M Nakagawa Y Yamadera R. Does the Endometrial Thickness on the Day of the Trigger Affect the Pregnancy Outcomes After Fresh Cleaved Embryo Transfer in the Clomiphene Citrate-Based Minimal Stimulation Cycle? Reprod Med Biol (2020) 19(2):151–7. doi: 10.1002/rmb2.12315 PMC7138937 32273820

[7] Crosby D O'Brien Y Glover L Martyn F Wingfield M. Influence of Body Mass Index on the Relationship Between Endometrial Thickness and Pregnancy Outcome in Single Blastocyst Frozen Embryo Transfer Cycles. Hum Fertil (Camb) (2020) 23(1):32–7. doi: 10.1080/14647273.2018.1504324 30221570

[8] Alpha Scientists in Reproductive Medicine and ESHRE Special Interest Group of Embryology. The Istanbul Consensus Workshop on Embryo Assessment: Proceedings of an Expert Meeting. Hum Reprod (2011) 266(6):1270–83. doi: 10.1093/humrep/der037 21502182

[9] Liu KE Hartman M Hartman A. Management of Thin Endometrium in Assisted Reproduction: A Clinical Practice Guideline From the Canadian Fertility and Andrology Society. Reprod BioMed Online (2019) 39(1):49–62. doi: 10.1016/j.rbmo.2019.02.013 31029557

[10] Hastie T Tibshirani R. Generalized Additive Models. Boca Raton: Chapman and Hall (1990).

[11] R Development Core Team. R: A Language and Environment for Statistical Computing. Vienna, Austria: R Foundation for Statistical Computing (2008)ISBN 3-900051-07-0.

[12] Liu KE Hartman M Hartman A Luo ZC Mahutte N. The Impact of a Thin Endometrial Lining on Fresh and Frozen-Thaw IVF Outcomes: An Analysis of Over 40 000 Embryo Transfers. Hum Reprod (2018) 33(10):1883–8. doi: 10.1093/humrep/dey281 PMC6145412 30239738

[13] Pan Y Hao G Wang Q Liu H Wang Z Jiang Q. Major Factors Affecting the Live Birth Rate After Frozen Embryo Transfer Among Young Women. Front Med (Lausanne) (2020). doi: 10.3389/fmed.2020.00094 PMC7105776 32266278

[14] Vaegter KK Lakic TG Olovsson M Berglund L Brodin T Holte J. Which Factors Are Most Predictive for Live Birth After *In Vitro* Fertilization and Intracytoplasmic Sperm Injection (IVF/ICSI) Treatments?Analysis of 100 Prospectively Recorded Variables in 8,400 IVF/ICSI Single-Embryo Transfers. Fertil Steril (2017) 1073(3):641–8.e2. doi: 10.1016/j.fertnstert.2016.12.005 28108009

[15] Broer SL van Disseldorp J Broeze KA Dolleman M Opmeer BC Bossuyt P. Added Value of Ovarian Reserve Testing on Patient Characteristics in the Prediction of Ovarian Response and Ongoing Pregnancy: An Individual Patient Data Approach. Hum Reprod Update (2013) 19:26–36. doi: 10.1093/humupd/dms041 23188168

[16] Li J Zhang F Sun B Dai S Yang Q Hu L. Lower Chromosomal Abnormality Frequencies in Miscarried Conceptuses From Frozen Blastocyst Transfers in ART. Hum Reprod (2021) 36(4):1146–56. doi: 10.1093/humrep/deaa352 33378532

[17] Glujovsky D Farquhar C Quinteiro Retamar AM Alvarez Sedo CR Blake D. Cleavage Stage Versus Blastocyst Stage Embryo Transfer in Assisted Reproductive Technology. Cochrane Database Syst Rev (2016) (6):CD002118. doi: 10.1002/14651858 27357126

[18] Shakerian B Turkgeldi E Yildiz S Keles I Ata B. Endometrial Thickness is Not Predictive for Live Birth After Embryo Transfer, Even Without a Cutoff. Fertil Steril (2021) 116(1):130–7. doi: 10.1016/j.fertnstert.2021.02.041 33812651

[19] Kasius A Smit JG Torrance HL Eijkemans MJ Mol BW Opmeer BC. Endometrial Thickness and Pregnancy Rates After IVF: A Systematic Review and Meta-Analysis. Hum Reprod Update (2014) 204(4):530–41. doi: 10.1016/j.rbmo.2021.04.007 24664156

[20] Gao G Cui X Li S Ding P Zhang S Zhang Y. Endometrial Thickness and IVF Cycle Outcomes: A Meta-Analysis. Reprod BioMed Online (2020) 40(1):124–33. doi: 10.1016/j.rbmo.2019.09.005 31786047

